# Poor preoperative performance at Clock Drawing Test is associated with postoperative decline in olfaction in older patients: an observational pilot study

**DOI:** 10.1186/s12871-023-02256-0

**Published:** 2023-08-30

**Authors:** Victoria Van Regemorter, Richard Coulie, Jordi Dollase, Mona Momeni, Alexandre Stouffs, Lisa Quenon, André Mouraux, Caroline Huart

**Affiliations:** 1 Department of Anesthesiology, Cliniques Universitaires Saint-Luc, Avenue Hippocrate 10, 1200 Brussels, Belgium; 2 Institute of Neuroscience, Université catholique de Louvain, Brussels, Belgium; 3 Department of Neurology, Cliniques Universitaires Saint-Luc, Brussels, Belgium; 4 Department of Otorhinolaryngology, Cliniques Universitaires Saint-Luc, Brussels, Belgium

**Keywords:** Olfaction, Clock Drawing Test, Aged, Preoperative cognitive function, Postoperative complications

## Abstract

**Background:**

Decline in olfaction may occur after general anesthesia, but the exact incidence and underlying physiopathology remain scarcely investigated. Olfactory dysfunction arises with aging and is known to be linked to cognitive impairment. In this pilot study, we evaluated the incidence of immediate postoperative decline in olfaction and its association with a preoperative cognitive test, performance at Clock Drawing Test (CDT), in a group of older patients.

**Methods:**

This pilot study is a sub-analysis of a prospective observational study. Patients ≥ 65 years old and scheduled for elective non-cardiac surgery under sevoflurane-based anesthesia were enrolled. CDT was part of the preoperative evaluation. We assessed olfaction on the day before and the day after surgery (between 16 and 26 h postoperatively) using the Sniffin’ Sticks 12-item identification test, which consists of pen-like devices displaying 12 different odors. Postoperative decline in olfaction was defined as a decrease of at least 1 standard deviation in the olfactory score.

**Results:**

We included a total of 93 patients, among whom 19 (20.4%) presented a postoperative decline in olfaction. The incidence of postoperative decline in olfaction was higher in the “CDT low-score” (score ≤ 5/8) group (11/34, 32.4%) than in the “CDT high-score” (score ≥ 6/8) group (8/58, 13.6%) (*P* = 0.030). Despite adjusting for confounding variables, CDT score remained independently associated with immediate postoperative decline in olfactory identification function (OR 0.67, 95% CI 0.48 to 0.94, *P* = 0.022).

**Conclusions:**

Postoperative decline in olfaction occurred in 20.4% of older patients and was associated with poor preoperative performance at CDT.

**Trial registration:**

This study was retrospectively registered on https://clinicaltrials.gov/ under the NCT04700891 number (principal investigator: Victoria Van Regemorter), in December 2020.

## Background

The sense of smell contributes to social communication, guides nutrition behavior and is crucial to detect daily life hazards [1]. Olfactory dysfunction may thus have a severe impact on health and quality of life. Incidence of olfactory dysfunction increases with age, as a result of several mechanisms: exposure to toxins or drugs, infection, trauma, but also brain aging and neurodegenerative diseases [2, 3].

Decline in olfactory function has been reported as a complication of general anesthesia for all types of surgeries, however its frequency and physiopathology remain largely unknown. Several reports have been published over the years, mainly describing isolated cases of patients with sudden postoperative onset of medium to long-lasting olfactory or gustatory dysfunction [4–8]. On the other hand, some small-sized studies have shown a possible detrimental effect of the use of sevoflurane compared to other anesthetic agents on immediate postoperative olfactory function [9–12]. In a recent study, age was correlated to poorer postoperative olfactory performances, thereby suggesting interactions between cognitive impairment, which is more prevalent with age, [13] and olfactory dysfunction [14].

To evaluate the incidence of immediate postoperative decline in olfaction and to address whether it is associated with preoperative cognitive performance, we performed a pilot study. We realized a sub-analysis of a previously published prospective observational study in older patients undergoing various types of elective non-cardiac surgery under sevoflurane-based anesthesia [15]. We hypothesized that preoperative performance at the Clock Drawing Test (CDT) – which engages multiple cognitive functions, including visuoconstructive abilities, executive function and semantic memory, [16] and is used for the preoperative assessment of cognition [17] would be related to postoperative decline in olfactory identification score. Moreover, we aimed at investigating the potential relationship between intraoperative administered drugs doses and worse postoperative olfaction.

## Methods

### Study population and protocol

This pilot study is a sub-analysis of a prospective observational study conducted at the Cliniques universitaires Saint-Luc (Brussels, Belgium) between July and October 2020 [15]. The whole study protocol was approved (2020/22JAN/050) by the institutional Ethics Committee of Cliniques universitaires Saint-Luc, Université catholique de Louvain, Brussels, Belgium (Chairperson J.-F. Maloteaux) on 3 March 2020. At that time, the authors were not aware of the recommendation to register prospective observational studies. It was therefore registered retrospectively on https://clinicaltrials.gov/ under the NCT04700891 number (principal investigator: Victoria Van Regemorter, https://beta.clinicaltrials.gov/study/NCT04700891), in December 2020. All participants gave their written informed consent. This manuscript adheres to the applicable STROBE guidelines.

The complete study protocol has been described previously [15]. The aims of the primary trial were to correlate preoperative olfactory identification function with frailty and cognitive performance, assessed by the CDT, as well as with postoperative outcome. Inclusion criteria were patients aged ≥ 65 years old who were scheduled for all minor, intermediate or major inpatient non-cardiac surgery under sevoflurane-based anesthesia. Head and neck surgery patients were not included since postoperative olfactory dysfunction may occur as a surgical complication. Exclusion criteria were as follows: history of neurological (including any type of diagnosed dementia) or psychiatric disorder, severe head trauma, chronic rhinosinusitis, post-infectious olfactory dysfunction or current acute upper respiratory tract infection. Any history of past or current COVID-19 infection was also an exclusion criterion, which was verified either by a PCR-test taken just before the surgery or by interrogating the patient about any related symptoms, especially, any recent change in smell or taste abilities.

We performed preoperative and postoperative olfactory assessments. Preoperative olfactory testing was realized on the day before surgery and was also accompanied by a frailty assessment including an evaluation of cognitive performance. Postoperative olfactory testing was realized on the day after the surgery, so that all patients took their test between 16 and 26 h after their surgery. In order to get the same conditions for pre- and postoperative tests, both were realized in the patient’s room. Three physicians were trained specifically together to administer the tests in the exact same way and always made sure to get a calm and uninterrupted testing session with the patients. None of the patients tested postoperatively were delirious when the olfactory test was carried out.

All patients received general anesthesia with intravenous induction followed by maintenance with sevoflurane. Induction agents were left to the discretion of the anesthesiologist in charge of the patient. Sevoflurane maintenance dose was adjusted by achieving end-tidal concentration equivalent to 1 age-adjusted MAC. All the patients received proper routine intraoperative care using an individualized approach depending on each patient’s comorbidities. Vasopressors were administered whenever needed to maintain adequate hemodynamics. Protective ventilation was administered to all patients and the target for end-tidal carbon dioxide ranged between 35 and 45 mmHg. Normothermia was maintained with forced-air warming or fluid warming systems. Patients were extubated at the end of the surgical procedure then transferred to the post-anesthesia care unit. We did not record any intra-or postoperative major event regarding hemodynamics or ventilation. No patient was admitted to the intensive care unit in the immediate postoperative period.

### Predictor variables

All patients had a preoperative frailty evaluation with the Edmonton Frail Scale, since constituting the primary outcome of the initial study [15]. In this sub-analysis, we focused on the Clock Drawing Test (CDT), which is part of the Edmonton Frail Scale, as preoperative cognitive testing. In this test, the circle of the clock was already provided, and the patients were asked to put in all the numbers, then to set the hands to 10 after 11 [18]. The CDT increases total Edmonton Frail Scale score by one point for minor spacing errors and by two points for other errors. However, to improve scoring accuracy, each clock was also scored separately from the Edmonton Frail Scale using Rouleau’s scale [19], in which 10 is the maximum score and represents the best performance. A score of ≤ 7/10 usually indicates significant cognitive impairment [20]. Indeed, normative data obtained for French-Quebec healthy older adults confirmed that the score of 7 represents at most the 10th percentile in the oldest, thus reflecting at least mild cognitive impairment [21]. Here, we removed the two points originally awarded for the adequate drawing of the clockface from both the total score and the cut-off. Patients were thus categorized in the “CDT high-score” group when their score was 6 or more or in the “CDT low-score” group when scoring ≤ 5/8.

Moreover, we recorded the doses of anesthetic as well as analgesic intravenous drugs administered intra- or postoperatively in our patients and suspected to have a potential influence on olfactory function [7, 22, 23]. Duration of surgery, reflecting duration of sevoflurane exposure, was also reported. Other information collected included demographic variables, education level and American Society of Anesthesiologists (ASA) physical status. Grades of the surgeries were classified according to the European Society of Anesthesiology cardioavascular assessment guidelines [24].

### Outcome variable

#### Olfactory function assessment

**Fig. 1:**
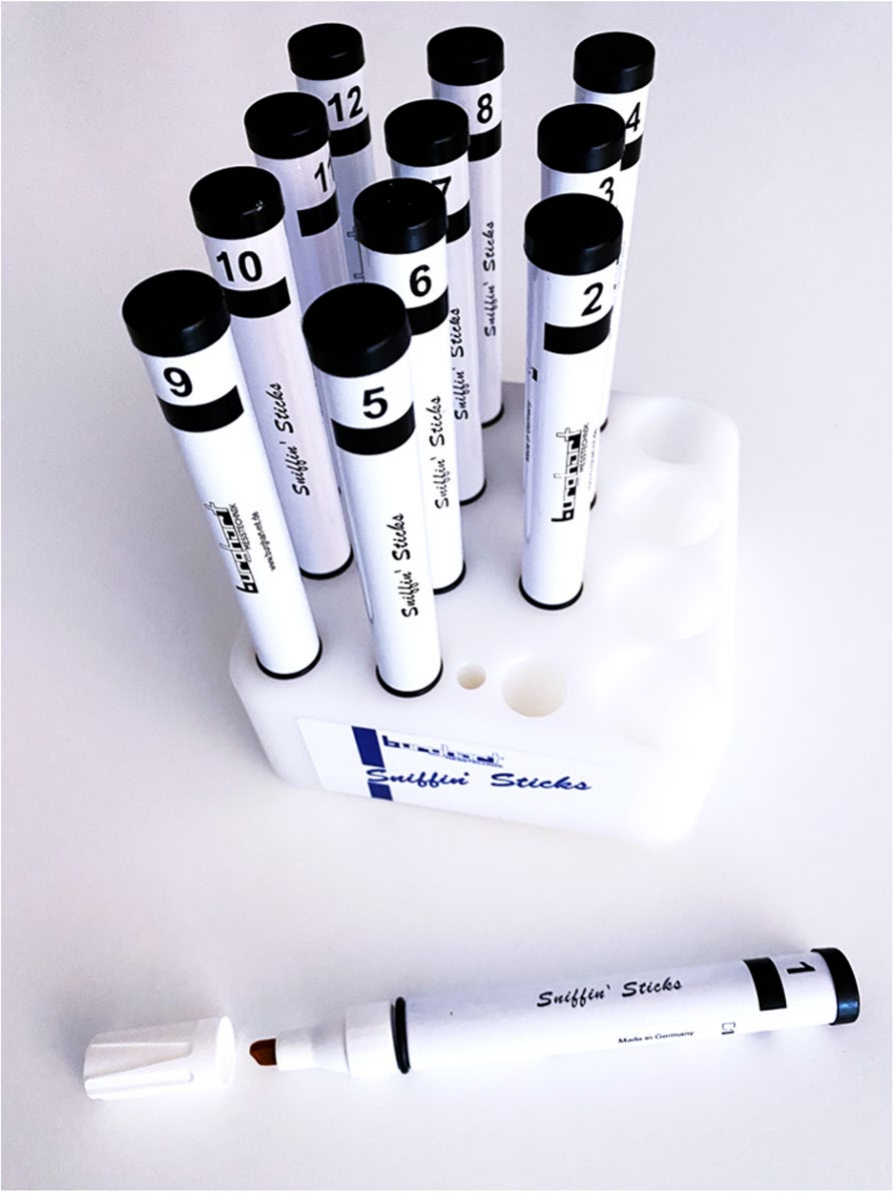
The Sniffin’ Sticks 12-item identification test (Burghart Messtechnik GmbH, Wedel, Germany). Legend: This test consists of 12 pen-like odor dispensing devices, each displaying a different odor. The patient is asked each time to make a forced choice from a list of 4 descriptors. Total olfactory score ranges from 0 to 12 points, where 12 is the best score

Olfactory function was evaluated with a validated psychophysical testing method that is the Sniffin’ Sticks 12-item identification test (Burghart Messtechnik GmbH, Wedel, Germany), which does not include the evaluation of the threshold and discrimination modalities of olfactory function. This olfactory identification test consists of pen-like odor dispensing devices displaying 12 different odors (see Fig. 1), namely orange, leather, cinnamon, peppermint, banana, lemon, licorice, coffee, cloves, pineapple, rose and fish. The pens were consecutively placed approximately 2 cm in front of the nostrils for approximately 3 s, and the patients were then asked to make a forced choice from lists of four descriptors each. The descriptors were written down on cards presented in front of the patient. For each correct answer, one point was awarded and points were added to obtain a final olfactory score ranging from 0 to 12. Since there is a 25% probability of a random correct answer for each pen, chance by itself would already provide on average a total score of 3 points out of 12. Patients scoring 3 or less at preoperative olfactory test were excluded.

The preoperative and postoperative olfactory identification tests were identical. To minimize test–retest bias, the patients were not told about their test results during the preoperative assessment. We calculated postoperative change in olfactory identification score as the numerical difference of postoperative olfactory identification score minus preoperative olfactory identification score. We then defined postoperative decline in olfactory identification score as a decrease of at least 2 points (corresponding to one standard deviation relative to the average change across all patients). Conversely, a better postoperative performance in olfactory identification score was characterized as an increase of at least 2 points (i.e. one standard deviation). A difference of zero or one point was considered unsignificant change.

### Statistical analysis

The main objectives were to assess the incidence of postoperative decline in olfactory identification function and to evaluate its association with preoperative performance at CDT, as cognitive testing. Furthermore, we analyzed the association of intraoperative drugs doses with postoperative decline in olfactory identification function. We did not realize any power analysis for the aims of this pilot study since it was considered exploratory. Data were analyzed using SPSS version 27.0. The Kolmogorov–Smirnov test was used to check the normality of the data. Continuous data were not normally distributed and were expressed as medians (interquartile range). Comparisons between the “CDT high-score” group and the “CDT low-score” group were realized with a Pearson χ^2^ test for nominal variables and with a Mann–Whitney U-test for continuous variables. We used univariable and multivariable binary logistic regression analyses to assess the relationship between CDT score and the presence of postoperative decline in olfactory identification score. For these regression analyses, we considered any potential confounding variables regarding olfactory function (age, gender) or postoperative cognitive function (level of education, ASA physical status, surgery grade). The secondary objective was to compare intraoperative drugs doses between the patients who presented a postoperative decline in olfactory identification function and the patients who did not. Continuous data were compared using a Mann–Whitney U-test. The presence of patient-controlled intravenous analgesia in the ward was compared with a Pearson χ^2^ test. Any *P* value < 0.05 was considered significant.

## Results

**Fig. 2:**
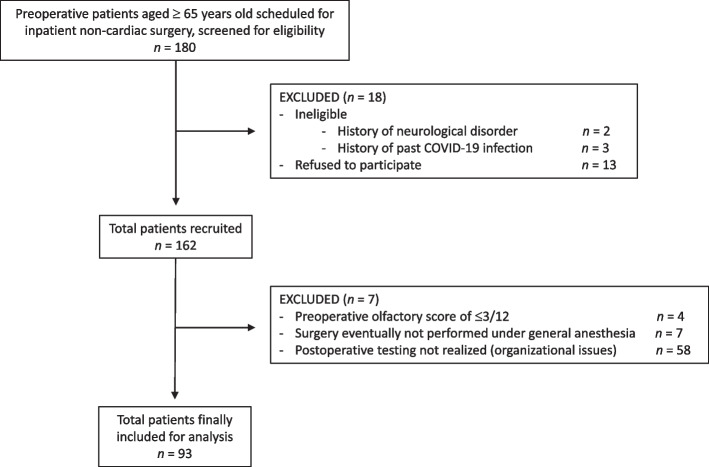
Study flowchart. Legend: This pilot study is a sub-analysis of a prospective observational study. Final sample size is 93 patients

**Fig. 3:**
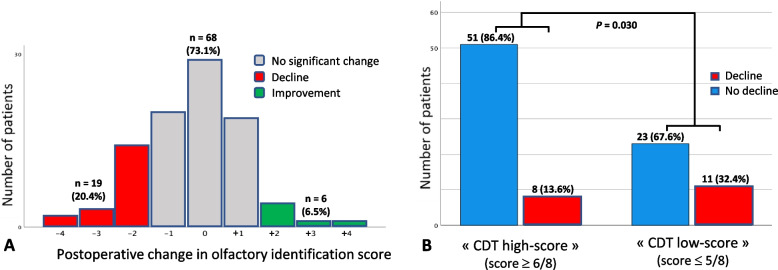
Postoperative change in olfactory identification score. Legend: **A** Individual distribution. The axis line of the histogram represents the numerical difference of postoperative minus preoperative olfactory identification score. **B** Comparison according to preoperative performance at Clock Drawing Test. The “Decline” category represents patients showing a postoperative decline in olfactory identification score of at least one standard deviation (corresponding to a 2 point decrease). The “No decline” category gathers patients presenting either no significant postoperative change or a postoperative increase in olfactory identification score. CDT, Clock Drawing Test

We finally included 93 patients for the purposes of this pilot study (see flowchart on Fig. 2). In total, when considering the whole cohort, median preoperative olfactory identification score was 10 [8,11] compared to 9 [8,11] after the surgery. Individual postoperative change in olfactory identification scores is detailed in Fig. 3A. Among all patients, 19 (20.4%) presented a postoperative decline in their olfactory identification, making from 2 to 4 more errors compared to their preoperative test. 68 patients (73.1%) did not significantly change their postoperative score. A better postoperative performance was only observed in 6 (6.5%) patients who increased their olfactory score by 2 to 4 points.

Baseline and operative characteristics of all the patients are summarized in Table 1. Median age was 72 years old [68,78] and women comprised 58.1% of the cohort. Patients were classified in two groups according to their preoperative performance at CDT, as described beforehand. Age, gender and body mass index were similar between the two groups. Among the “CDT low-score” group, 52.9% of patients (18/34) had a lower education level whereas they represented only 32.2% (19/40) of the “CDT high-score” group (*P* = 0.049). ASA physical status and grades of surgeries also did not differ significantly.

**Table 1:** Baseline and perioperative characteristics of patients according to their performance at Clock Drawing Test

Characteristics	“CDT high-score” group (*n* = 59)	“CDT low-score” group (*n* = 34)	*P* Value
Age, year	72.0 [68.0, 78.0]	72.5 [69.0, 77.0]	0.933
Female, n (%)	33 (55.9)	21 (61.8)	0.583
Body mass index, kg.m^−2^	26.7 [23.7, 30.8]	27.2 [24.1, 30.1]	0.854
Education level, n (%)			0.049
Lower (≤ 9 years)	19 (32.2)	18 (52.9)	
Higher (> 9 years)	40 (67.8)	16 (47.1)	
ASA physical status, n (%)			0.277
I—II	35 (59.3)	24 (70.6)	
III	24 (40.7)	10 (29.4)	
Grade of surgery, n (%)			0.912
Minor	15 (25.4)	9 (26.5)	
Intermediate / Major	43 (72.9) / 1 (1.7)	25 (73.5) / 0 (0.0)	

Data are expressed as median [IQR] or number (%). *CDT* Clock Drawing Test, *ASA* American Society of Anesthesiologists

Preoperative performance at the CDT was associated with postoperative decline in olfactory identification (χ^2^ (1) = 4.69, *P* = 0.030). The results are shown in Fig. 3B. 32.4% of the patients in the “CDT low-score” group (11/34) presented a postoperative decline in olfactory identification against only 13.6% (8/59) in the “CDT high-score” group.

Univariable and multivariable logistic regression analyses were performed for the CDT score and other potential confounding variables to evaluate their association with the presence of postoperative decline in olfaction (Table 2). After adjustment for age, gender, education level, ASA physical status and grade of surgery, CDT score remained a significant predictor variable. The higher the CDT score was, the lesser were the odds of presenting a postoperative decline in olfactory identification score (OR 0.67, 95% CI 0.48 to 0.94, *P* = 0.022). In the univariable analysis, ASA physical status III was the only other variable to be significantly associated with the presence of postoperative decline in olfaction (OR 3.05, 95% CI 1.08 to 8.59, *P* = 0.035). However, statistical significance was lost in the multivariable model.

**Table 2:** Univariable and multivariable logistic regression analysis for the prediction of a postoperative decline in olfactory identification score

Variable	Univariable			Multivariable		
	OR	95% CI	*P* Value	OR	95% CI	*P* Value
CDT score	0.72	0.53–0.99	0.040	0.67	0.48–0.94	0.022
Age	1.03	0.95–1.11	0.472	1.03	0.95–1.12	0.510
Female	0.76	0.28–2.09	0.591	0.86	0.27–2.74	0.803
Lower education level (≤ 9 years)	0.86	0.30–2.42	0.769	0.70	0.22–2.25	0.550
ASA physical status III	3.05	1.08–8.59	0.035	3.21	1.00–10.30	0.051
Intermediate or major surgery	0.70	0.23–2.10	0.520	0.87	0.26–2.89	0.822

*OR* Odds Ratio, *CI* Confidence Interval, *CDT* Clock Drawing Test, *ASA* American Society of Anesthesiologists

We also aimed at analyzing the association between drugs and postoperative decline in olfactory identification score. Overall, median duration of sevoflurane exposure was 146 min (ranging from 103 to 213 min) and was similar in patients with or without a postoperative decline in olfaction. Sufentanil, ketamine, midazolam and rocuronium doses did not differ between the two groups of patients. On the contrary, median propofol dose (mg/kg) was significantly higher in patients suffering from a postoperative decline in olfactory identification score (2.03 IQR [1.54 to 2.76] vs 1.67 IQR [1.21 to 2.02], *P* = 0.025). Postoperative morphine consumption was not statistically different between both groups.

## Discussion

The results of this pilot study in a group of older patients undergoing non-cardiac surgery under general anesthesia with sevoflurane show that 20.4% of patients experience a decline in their olfactory identification function on the first postoperative day compared to their preoperative score. Hernandez et al. analyzed changes in global olfactory function (threshold, discrimination and identification modalities) in the perioperative context in a group of 73 patients [14]. In contrast to our results, they had a worse postoperative global performance of olfactory function in only 5% of their cohort. This difference might be explained partly by the fact that among their 73 patients, only 62 received general anesthesia. Otherwise, their study population was younger (mean age of 51 years old). Interestingly, they observed an overall slight increase in postoperative mean olfactory identification score, which they explained by the known test–retest effect [25]. On the contrary, concerning the postoperative olfactory threshold test, they found that the positive test–retest effect decreased with age. Although we used an olfactory identification test, this age effect could contribute to the greater postoperative decline in our older cohort. Overall, we show a decrease in median olfactory identification score on the first postoperative day, which corroborates existing studies showing similar results either at 3 hours [9–11] or, using the discrimination modality, within 15 hours [12] postoperatively. In two of them, olfactory results were retested again at least 3 days postoperatively and came back to baseline [10, 11]. In the study from Hernandez and colleagues, postoperative testing occurred later (6 postoperative days on average) and this could have also contributed to the discrepancy of their results [14].

Poor preoperative performance at the CDT was independently associated with immediate postoperative decline in olfactory identification function, even after adjusting for potential confounding variables. Olfactory identification assessment is a quite simple test, which had already been taken the day before the surgery in our patients, but might still constitute a cognitively demanding task. Of note, it requires both a neurological pathway to “sense” the odor but also higher order cognitive abilities – in particular semantic and executive functions – to identify and eventually name the odor [26, 27]. Besides, the results emerging from our primary study showed a clear correlation between preoperative olfactory dysfunction and poor performance at CDT, also supporting the close relationship between olfaction and cognition [15]. Yet, poor performance at CDT before surgery has been shown to increase the risk of developing postoperative delirium [28, 29]. The stronger reduction in olfactory identification performance on the day following general anesthesia in poor CDT performers at baseline could thus merely reflect a transient decrease in postoperative cognitive abilities, occurring more often in patients with preoperative altered cognition.

Being classified as ASA physical status III has already been demonstrated to be an independent predictor of postoperative neurocognitive disorder in older patients [30–32]. Here, we show an association with worst postoperative olfactory function, though narrowly failing to reach the significance level in the multivariable model, likely because of a lack of power. Therefore, our findings may again support the hypothesis of postoperative olfactory performance being related to postoperative cognitive capacities. Without any pretension to replace a more complete cognitive examination (e.g., the Montreal Cognitive Assessment [33]), olfactory identification testing could potentially constitute a simple and quick clinical bedside tool to assess perioperative cognition.

Not surprisingly, we noted a link between a lower education level and worse performance at CDT. Preoperative cognitive testing is known to be influenced negatively by a low level of education, [34] and this was specifically shown for the CDT [35]. Nevertheless, lower education level was not associated with postoperative decline in olfaction and did not seem to affect the association with CDT score in the multivariable analysis.

In our study, intraoperative propofol doses were statistically higher in patients with a postoperative decline in olfactory identification function. Propofol has been incriminated in a few case reports as being responsible for postoperative olfactory dysfunction through its activation of gamma-aminobutyric acid type A (GABA_A_) receptors [7, 8], the latter being abundantly found in olfactory-related brain regions [36]. Beside the absence of real evidence supporting this, the difference of less than 0.5 mg/kg found between our two groups does not seem clinically significant. As to ketamine, which is thought to influence postoperative cognition, [37] the relatively small doses administered in the present cohort did not affect the results. Whether sevoflurane impacts negatively postoperative olfactory function still remains a matter of debate. Our data show that this potential effect, if any, is not related to the duration of exposure.

This study suffers from some limitations. First, this study was exploratory and was a sub-analysis of a larger prospective study, therefore power calculations were not realized specifically for the present aims. In addition, this study was registered retrospectively due to unawareness of registration policy. Second, preoperative cognitive function was evaluated on the sole basis of the CDT [16]. But, despite being neither time nor resource consuming, it does not assess all these cognitive domains with the same precision as a complete neurocognitive battery. Moreover, the method of removing from the total CDT score the 2 points originally allocated for the drawing of the circle has never been validated. Similarly, olfactory testing was limited to the identification modality without including an evaluation of olfactory detection thresholds and discrimination performance. Third, the timing of the postoperative olfactory identification test had a 10-h range which may have lowered the precision of our observations. Also, performing a second more delayed postoperative assessment of olfaction could have shown whether the observed decline in olfactory identification remained over time. Fourth, pain or anxiety inherent to the upcoming surgery might have had an influence on patients’ performance at preoperative testing. Fifth, we did not collect intraoperative burst suppression data and depth of anesthesia monitoring was not used. This means we did not analyze anesthesia depth and thereby its possible impact on postoperative cognition.

## Conclusions

In summary, this pilot study shows a decline in immediate postoperative olfactory identification function in around 20% of older patients scheduled for elective non-cardiac surgery under sevoflurane-based anesthesia. This was associated with poor preoperative performance at the CDT. Whether this decline in postoperative olfaction may be related to poor postoperative cognitive abilities and whether it may persist over time deserve future attention. Larger prospective studies with more comprehensive perioperative cognitive and olfactory testing should bring more insights to these preliminary data.

## Data Availability

The datasets used and analyzed during the current study are available from the corresponding author (Victoria Van Regemorter) on reasonable request.

## Abbreviations

CDT: Clock Drawing Test
ASA: American Society of Anesthesiologists
